# Cervicovaginal microbiome alters transcriptomic and epigenomic signatures across cervicovaginal epithelial barriers

**DOI:** 10.21203/rs.3.rs-6171614/v1

**Published:** 2025-05-07

**Authors:** Lauren Anton, Ana G. Cristancho, Briana Ferguson, Jacques Ravel, Michal A. Elovitz

**Affiliations:** University of Pennsylvania; Children’s Hospital of Philadelphia, University of Pennsylvania; University of Pennsylvania; University of Maryland School of Medicine; Icahn School of Medicine at Mount Sinai

**Keywords:** cervix, epithelial cells, Lactobacillus crispatus, Gardnerella vaginalis, RNA-seq, anti-microbial peptides, ATAC-seq, chromatin, women’s health

## Abstract

**Background:**

The cervicovaginal microbiome plays a critical role in women’s health, with microbial communities dominated by *Lactobacillus* species considered optimal. In contrast, the depletion of lactobacilli and the presence of a diverse array of strict and facultative anaerobes, such as *Gardnerella vaginalis*, have been linked with adverse reproductive outcomes. Despite these associations, the molecular mechanisms by which host-microbial interactions modulate cervical and vaginal epithelial function remains poorly understood.

**Results:**

In this study, we used RNA sequencing to characterize the transcriptional response of cervicovaginal epithelial cells exposed to the culture supernatants of common vaginal bacteria. Our findings revealed that *G. vaginalis* culture supernatants upregulate genes associated with an activated innate immune response and increased cell death. Conversely, *Lactobacillus crispatus* culture supernatants induced transcriptional changes indicative of epigenomic modeling in ectocervical epithelial cells. Epigenomic modification by *L. crispatus*, was confirmed by ATAC-sequencing, which demonstrated reduced chromatin accessibility.

**Conclusions:**

These results provide new insights into host-microbe interactions within the lower reproductive tract and suggests that modulating the vaginal microbiome could offer innovative therapeutic strategies to improve reproductive health.

## Introduction

The female lower reproductive tract is a complex ecosystem comprised of host epithelial and immune cells, a microbiome and a complex mixture of metabolites [1]. Over the past decade, the cervicovaginal microbiome has become the focus of extensive research due to its intricate and integral role in reproductive health and disease. High throughput 16S rRNA gene amplicon sequencing has allowed detailed characterization of vaginal microbiota composition in both pregnant and non-pregnant individuals [2–4]. Traditionally, the vaginal microbiome has been defined by the presence or absence of *Lactobacillus* species [2, 5, 6]. Cervicovaginal microbiomes dominated by *Lactobacillus* species are generally considered optimal and are associated with positive reproductive health outcomes. In contrast, microbiomes lacking lactobacilli and comprising a wide array of strict and facultative anaerobes have been linked to a range of adverse gynecological and reproductive outcomes including infertility [7, 8], sexually transmitted infections (STIs) (*e.g.*, human papilloma virus [HPV] [9] and human immunodeficiency virus [HIV] [10]), and pregnancy complications such as preterm birth [11, 12]. While the cervicovaginal microbiome is less taxonomically diverse than those at some body sites such as the gut, the presence of con-specific genotypes cohabitating within these microbiomes adds to their complexity [13]. Importantly, not all women with lactobacilli-deficient, anaerobe-rich cervicovaginal microbiomes experience negative clinical outcomes. This fact suggests that the contribution of a suboptimal cervicovaginal microbiome to adverse reproductive outcomes may depend on host-microbe or microbe-microbe interactions within the cervicovaginal space. Understanding the complexity of these interactions is essential to elucidate the precise mechanisms by which vaginal bacteria modulate host epithelial functions and contribute to adverse health outcomes.

The vaginal microbiome can interact with all epithelial barriers in the cervicovaginal space. These epithelial barriers are unique because the cells lining this space have distinct embryological origins, resulting in specialized cell functions, such as mucus production in the cervix [14–17]. As the primary site of entry for pathogens, the integrity of this barrier is critical, and its disruption is associated with increased susceptibility to STIs (*e.g.*, chlamydia, gonorrhea, HIV) [18, 19]. Metabolites, proteins and other products from common vaginal bacteria, including *Gardnerella vaginalis* and *Lactobacillus crispatus*, have been shown to exert distinct biological effects in the cervicovaginal space. Culture supernatants from non-optimal bacteria, such as *G. vaginalis*, trigger innate immune responses in cervicovaginal epithelial cells, a process thought to protect the epithelial barrier [20–23]. In contrast, lactic acid, a metabolite produced by *Lactobacillus* species, helps maintain an acidic vaginal pH [24], exhibits significant anti-microbial and anti-viral activity against Bacterial Vaginosis (BV)-associated bacteria [25, 26] and HIV [27, 28], modulates inflammatory responses [29–31] and increase cervical epithelial barrier integrity [32, 33]. Despite these findings, the molecular mechanisms underlying the beneficial and/or harmful effects of these vaginal bacteria remain poorly understood.

Elucidating the molecular mechanisms by which cervicovaginal microbiomes modulate host responses in the lower reproductive tract is critical to understanding their role in reproductive health and disease. The objectives of this study were to: 1) use unbiased discovery-based RNA-sequencing to identify genes and functional pathways in cervical and vaginal epithelial cells altered by exposure to *G. vaginalis* or *L. crispatus* culture supernatants; 2) characterize the immune pathways activated by *G. vaginalis* culture supernatants; and 3) uncover the molecular mechanisms by which *L. crispatus* culture supernatants optimize cervical and vaginal epithelial barriers.

## Results

### Cervicovaginal epithelial cell gene transcription and associated functional pathways are differentially modulated by L. crispatus and G. vaginalis culture supernatants

**Figure 1:**
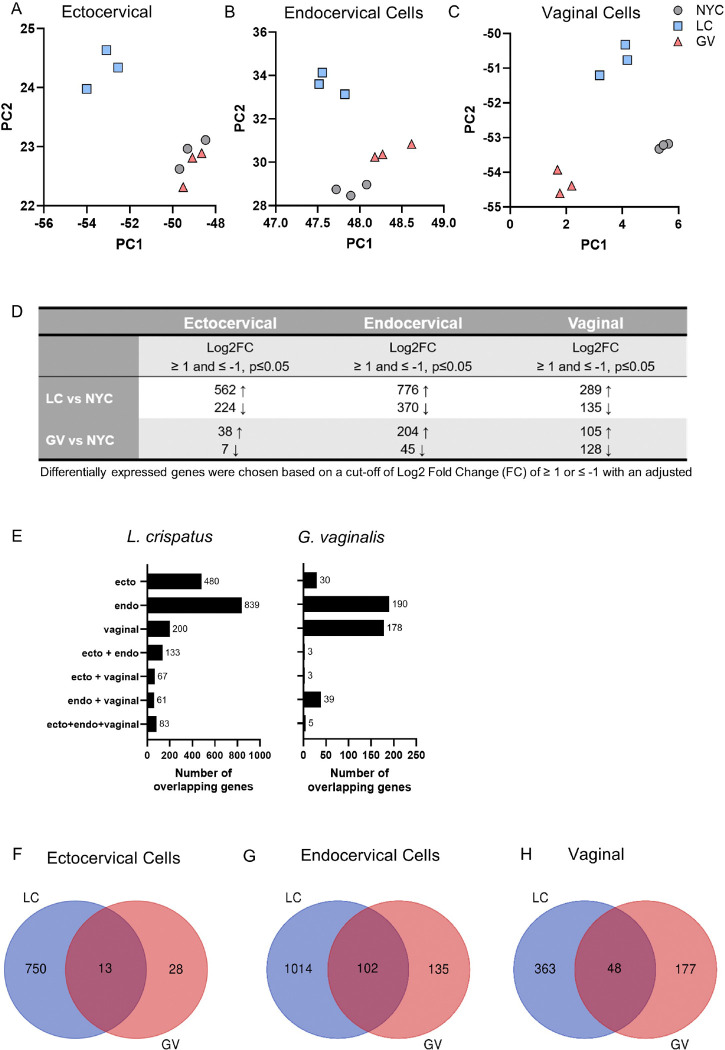
RNA-seq identified differentially expressed genes in cervicovaginal epithelial cells after 24 hr exposure to culture supernatants from *L. crispatus* or *G. vaginalis*. Principle component analysis (PCA) plots showing gene expression modulation in ectocervical (A), endocervical (B) and vaginal (C) cells exposed to either *G. vaginalis* or *L. crispatus* culture supernatants (vs NYCIII control). The total number of differentially expressed genes (adj. p<0.05, Log2FoldChange ≥ 1 and ≤ −1) in each exposure group by cell line (D). The number of overlapping differentially expressed genes between cervicovaginal cell types for each bacterial exposure (E-J). The number of overlapping differentially expressed genes between bacterial exposures within each cervicovaginal cell types (F-H).

Exposure of cervical and vaginal epithelial cells to *L. crispatus* or *G. vaginalis* bacteria-free culture supernatants resulted in significant differences in gene expression profiles. Principal component analysis (PCA) plots revealed distinct clustering of gene expression profiles by bacterial exposure across ectocervical, endocervical and vaginal epithelial cells (Fig. 1A–C). Notably, exposure to *L. crispatus* culture supernatant showed the most pronounced separation from the NYCIII bacterial culture medium control (Fig. 1A–C). For *G. vaginalis* culture supernatants, clustering patterns were distinct from the NYCIII control in endocervical and vaginal cells but not in ectocervical cells, highlighting cell type-specific responses (Fig. 1A–C). To ensure robust identification of differentially expressed genes, we applied stringent criteria: an adjusted p-value ≤ 0.05 and a log2 fold change ≥ 1 or ≤ −1 (Fig. 1D, Supplemental Tables 1 and 2). Using these thresholds, we found that the number of differentially expressed genes was highest after exposure to *L. crispatus* culture supernatants, followed by *G. vaginalis* (Fig. 1D). A minority of these genes overlapped between cell types for each bacterial culture supernatant exposure (Fig. 1E). For *G. vaginalis*, endocervical and vaginal epithelial cells exhibited the highest number of cell-specific differentially expressed genes, while *L. crispatus* culture supernatant elicited the highest number of cell-specific genes in ectocervical and endocervical epithelial cells (Fig. 1E, Supplemental Table 3). Within each cell type, we performed comparisons to identify commonly modulated gene expression across culture supernatant exposures (Fig. 1F–H, Supplemental Table 4). This analysis revealed unique genes differentially regulated by *G. vaginalis* and *L. crispatus* culture supernatants, demonstrating specific molecular effects of supernatant exposures on cervicovaginal epithelial cell transcription (Supplemental Table 5).

**Figure 2:**
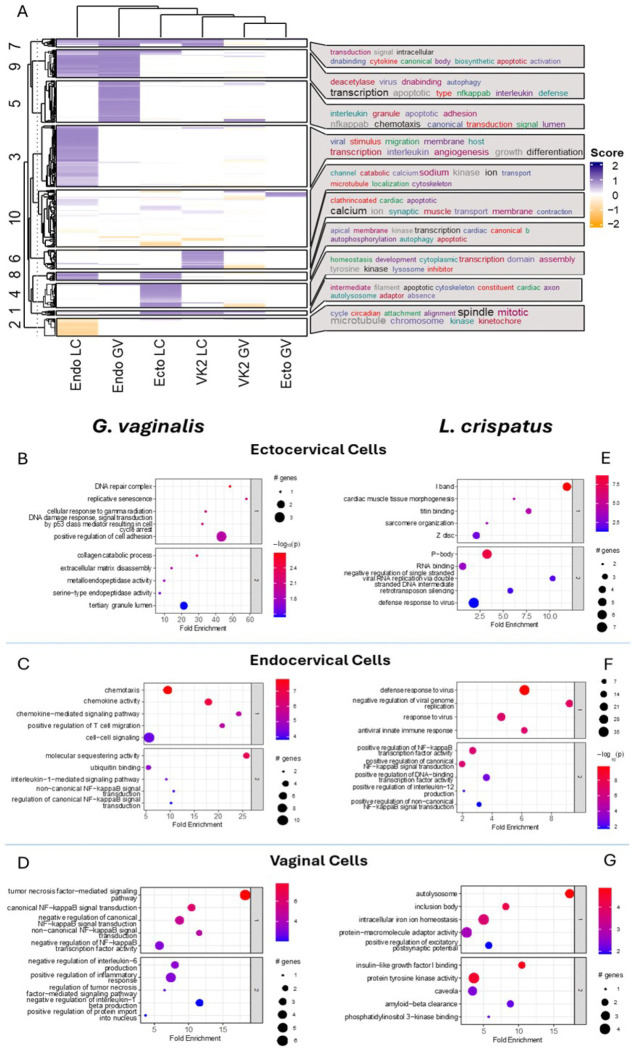
Differential clustering of significant differentially expressed genes (adj. p<0.05, Log2FoldChange ≥ 1 and ≤ −1) between exposure groups and across cervicovaginal cell lines reveal modulation of functional pathways (A). Functional pathway analysis (B-G) of RNA-seq data for ectocervical (B, E), endocervical (C, F) and vaginal (D, G) epithelial cells exposed to *G. vaginalis* or *L. crispatus*culture supernatants.

We conducted gene ontology (GO) analysis of upregulated and downregulated genes for each cell line and culture supernatant exposure combination to investigate these transcriptional differences further. This analysis uncovered overlapping or distinct cellular responses to bacterial culture supernatant exposures (Supplemental Tables 6a-i) [34, 35]. An aggregate dysregulation score was calculated for all affected pathways per sample, and averages for each cell line were visualized as heatmaps to reflect the diversity of gene expression changes between bacterial culture supernatant exposures (Fig. 2A) [34]. Unsupervised clustering of GO terms revealed specific trends, and a word cloud was generated to highlight the top GO terms associated with each cluster [36, 37]. Notably, themes of inflammatory and transcriptional dysregulation emerged. To identify the most critically dysregulated pathways in each cell type, we clustered GO term differences by cell line and bacterial supernatant exposure, focusing on the top clusters for further investigation [34]. Genes upregulated by *G. vaginalis* culture supernatants were predominantly associated with inflammation functional pathways (Fig. 2B–D). In contrast, exposure to *L. crispatus* culture supernatant was associated with modulation of transcriptional pathways, including histone modifications, RNA polymerase II and DNA binding (Fig. 2A, 2E–G).

### G. vaginalis, but not L. crispatus, culture supernatant dysregulates the innate immune response

**Figure 3:**
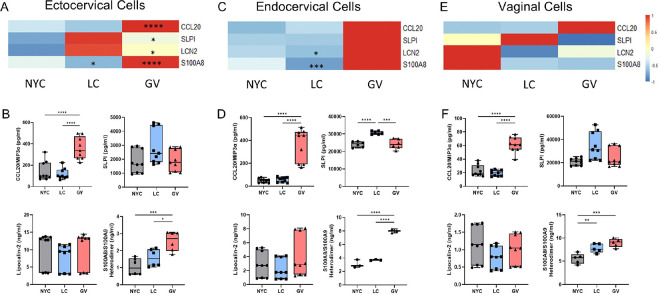
Anti-microbial peptide gene expression and proteins are significantly increased after cervicovaginal cell exposure to *G. vaginalis* culture supernatants. RNA-sequencing identified CCL20, SLPI, LCN2 and S100A8 as being significantly upregulated by *G. vaginalis* culture supernatants in ectocervical (A), endocervical (C) and vaginal (E) epithelial cells. Heatmaps represent differences in normalized reads across rows with significance denoted by asterisks. ELISAs further identified that CCL20 and S100A8 protein levels were increased after *G. vaginalis* culture supernatant exposure (B, D, F). Values are mean ± SEM. Asterisks over solid lines represent comparisons between control (NYC) and treatment groups. *p<0.05, **p<0.01, ***p<0.001, ****p<0.0001.

Exposure of cervicovaginal cells to *G. vaginalis* culture supernatants led to the differential expression of genes significantly associated with innate inflammation-related functional pathways (Fig. 2A, 2B–2D). Canonical NF-kB pathway genes encoding for multiple chemokines and cytokines such as IL-8, IL-6 and TNFα (Supplemental Table 2) were upregulated, consistent with prior findings by our group and others [22, 38–40]. Additionally, several anti-microbial peptides (AMPs), key components of the innate immune response, were upregulated in cervicovaginal epithelial cells following exposure to *G. vaginalis* culture supernatant. These included Chemokine Ligand 20 (CCL20), Secretory Leukocyte Peptidase Inhibitor (SLPI), Lipocalin 2 (LCN2) and S100 Calcium Binding Protein 8 (S100A8/A9, Calgranulin). Of these, CCL20 was the only gene consistently upregulated across all three cell lines (adjusted p < 0.05, Fig. 3A, 3C, 3E, Supplemental Table 7). In ectocervical and endocervical cells, all four AMP genes were significantly upregulated following exposure to *G. vaginalis* culture supernatant (adjusted p < 0.05, Fig. 3A and C, Supplemental Table 7). However, in vaginal epithelial cells, only CCL20 was upregulated under the same conditions (adjusted p < 0.05, Fig. 3E, Supplemental Table 7).

Protein-level analysis via ELISA confirmed the overexpression of CCL20 and S100A8 (both p < 0.05) after exposure to *G. vaginalis* culture supernatants. In contrast, protein levels of SLPI or LCN2 remained unchanged despite their transcriptional upregulation (Fig. 3B, 3D, 3F). Furthermore, *G. vaginalis* culture supernatants increased cell death in ectocervical and endocervical cells but not vaginal epithelial cells. In comparison, *L. crispatus* culture supernatant had no effect on cell death (Supplemental Fig. 1).

Exposure to *L. crispatus* culture supernatants showed distinct effects on AMP gene expression. The gene expression of S100A8 was significantly reduced across all three cell lines (p < 0.001, Fig. 3A, 3C, 3E, Supplemental Table 7). Additionally, LCN2 was downregulated in endocervical and vaginal epithelial cells (p < 0.0001, Fig. 3C and 3E, Supplemental Table 7), while CCL20 gene expression was decreased specifically in endocervical cells (p < 0.0001, Fig. 3C, Supplemental Table 7). In ectocervical cells, however, SLPI and LCN2 gene expression were increased (p < 0.0001, Fig. 3A, Supplemental Table 7). Despite these transcriptional changes, exposure to *L. crispatus* culture supernatant was not associated with consistent changes in AMP protein levels (Fig. 3B, 3D and 3F) across cell lines.

### L. crispatus culture supernatants alter chromatin accessibility

Exposure to *L. crispatus* culture supernatants resulted in significant changes in the expression of genes related to histone and transcriptional regulation in cervical and vaginal epithelial cells (Fig. 2A, 2E–G). To investigate whether these gene expression changes were associated with alterations in chromatin accessibility, we performed assay for transposase-accessible chromatin high throughput sequencing (ATAC-seq) [41–43].

The number and percentage of aligned and unaligned sequence reads were consistent across all three cell types (Supplemental Fig. 2). However, ectocervical epithelial cells exhibited lower transcription start site (TSS) enrichment scores despite showing similar quality control characteristics in alignment quality (Supplemental Fig. 3). A strong correlation in normalized sequence read counts between conditions was observed for each cell type, indicating high-quality samples (Supplemental Fig. 4).

**Figure 4:**
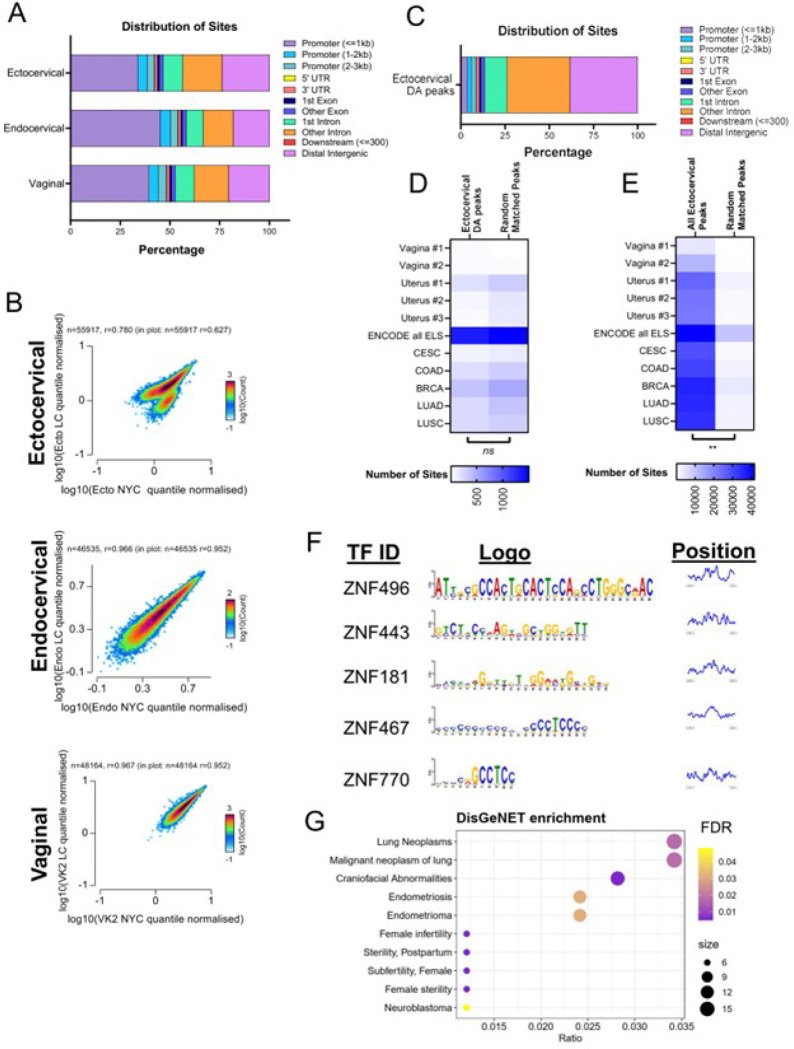
Chromatin accessibility was disrupted primarily in ectocervical cells exposed to *L. crispatus* supernatants. (A) Distribution of consensus sites by percentage. (B) Scatterplot of normalized counts between NYC and *L. crispatus*supernatant treatment by cell type-specific consensus peaks. (C) Distribution of differentially accessible sites in ectocervical cells across the genome. (D) Overlap of differentially accessible sites and matched number of random sites (E) all ectocervical peaks and matched number of random sites with published sites from ENCODE and multiple primary cancer specimens. (F) Motif analysis of downregulated differentially accessible sites with motif logo and graph of positional distribution based on the center of the peak of the top 5 motifs. (G) Bubble chart of DisGeNET enrichment of transcription factors identified by the motif analysis. DA: Differential Accessibility; ELS: Enhancer-like signatures; CESC: Cervical Squamous Cell Carcinoma; COAD: Colon Adenocarcinoma; BRCA: Breast Invasive Carcinoma; LUAD: Lung Adenocarcinoma; LUSC: Lung Squamous Cell Carcinoma.

A consensus peak set was obtained for each cell type (Ecto: 55,917 peaks, Endo: 46,535 peaks, VK2: 48,164 peaks). As expected, the majority of open chromatin peaks across all cell types were located in proximal promoter regions (< 1 kb) or intergenic regions (Fig. 4A). To assess cell-specific differences in chromatin organization, we compared normalized sequence read counts across genomic regions. While no differences were observed in quantile-normalized counts between TSS and gene bodies for each cell type (Supplemental Fig. 5), ectocervical epithelial cells uniquely exhibited two distinct clusters of chromatin accessibility in consensus peak regions (Fig. 4B). This clustering was not observed in endocervical and vaginal epithelial cells (Fig. 4B). Based on these findings and the pronounced epigenetic shifts detected in ectocervical epithelial cells via RNA sequencing, subsequent ATAC-seq analyses focused on ectocervical cells.

Tests for regions of differential accessibility between the treatment conditions were performed in the cell type-specific consensus peak sites (Supplemental Tables 8a-c). Differential accessibility analysis revealed 8,147 regions with altered chromatin accessibility in *L. crispatus* supernatant-treated ectocervical cells, with 8,125 regions showing decreased accessibility and only 22 regions showing increased accessibility. In contrast, endocervical and vaginal cells exhibited far fewer differentially regulated sites (21 and 109 total sites, respectively). In ectocervical cells, regions with decreased accessibility were predominantly located in distal intergenic and intronic regions, with a corresponding reduction in proximal promoter sites (Fig. 4C). Genes neighboring these differentially accessible regions overlapped significantly with those identified as differentially expressed by RNA-sequencing (683/762 genes) [44–46].

To explore the functional relevance of these findings, we assessed whether differentially accessible regions were enriched in tissue-specific regulatory elements or enhancer regions identified by the Encyclopedia of DNA Elements (ENCODE) Project in primary tissues or open accessibility regions identified in different primary cancer specimens [47–49]. Surprisingly, little overlap was observed between differentially accessible regions in ectocervical cells and putative enhancer regions (Fig. 4D, Chi-squared test for trend *p* = 0.8334). This lack of overlap may reflect the absence of primary cervical tissue specimens in the ENCODE dataset, as comparisons were limited to vaginal and uterine samples. In contrast, all accessible chromatin regions in ectocervical epithelial cells showed substantial overlap with published likely enhancer regions (Fig. 4E, Chi-squared test for trend *p* = 0.0028).

Motif analysis of downregulated differentially accessible sites identified enrichment for 497 transcription factor motifs with an FDR of 0.05 (Supplemental Table 9, Fig. 4F). The top five motifs were centrally positioned within the peaks, consistent with potential true transcription factor binding [50]. Gene-disease enrichment analysis of these transcription factors revealed associations with pathways related to neoplasms, endometriosis and infertility, conditions potentially mitigated by *Lactobacillus-*dominated microbiota (Fig. 4G) [51, 52]. Specificity analysis using random transcription factor lists confirmed these findings, as random motifs predominantly enriched for unrelated pathways, such as craniofacial anomalies (Supplemental Fig. 6).

## Discussion

Host-microbe interactions are critical determinants of health and disease across multiple biological systems. This study sheds light on unique molecular mechanisms underlying host-microbe interactions within the cervicovaginal space, addressing a significant gap in our understanding of reproductive health. Our findings reveal that *G. vaginalis*, a facultative anaerobic bacteria associated with many gynecological disorders, including STIs [9, 10], cervical cancer [53, 54], infertility [7, 8], and preterm birth [4, 12], induces diverse immune pathways, dysregulates the innate immune response, and increases epithelial cell death. In contrast, *L. crispatus*, a key species in optimal vaginal microbiomes, promotes epigenetic modifications in ectocervical cells without inducing cell death. Together, these findings highlight the complexity of host-microbe interactions in the lower reproductive tract and reveal distinct molecular pathways by which optimal and non-optimal bacteria contribute to reproductive health and disease.

While some studies have characterized cervicovaginal microbiomes using high-throughput sequencing technologies [33, 55, 56], few have investigated the host transcriptional and functional pathways altered by host-microbe interactions in different cervicovaginal epithelial cell types critical to the function of the lower reproductive tract. Our RNA-seq results demonstrate that *G. vaginalis* and *L. crispatus* culture supernatants modulate distinct host genes and functional pathways in a cell-type-specific manner. These findings reflect the distinct functional diversity of epithelial barrier cells in the lower genital tract. Notably, each cervicovaginal epithelial cell type exhibited unique transcriptomic signatures in response to bacterial culture supernatant exposure, suggesting that specific tissue microenvironments can contribute to the varied reproductive outcomes observed *in vivo.* Understanding the microbial transcriptional activity within these distinct epithelial niches is essential to elucidating microbial functions, and not simply their presence, that drive host responses.

Human studies have shown positive correlations between a pro-inflammatory state and the presence of an anaerobe-rich cervicovaginal microbiome [4, 22, 57–60]. For example, vaginal swabs from Kenyan and Ugandan women with non-optimal microbiomes revealed an upregulation of cytokines involved in the innate immune response [58]. Consistent with these findings, our RNA sequencing results show that exposure to *G. vaginalis* culture supernatant upregulates genes involved in innate immune signaling pathways (cytokines/chemokines), increases anti-microbial peptides (AMPs), and induces cell death in cervicovaginal epithelial cells. While the inflammatory response to *G. vaginalis* has been previously linked to cellular damage and death [22, 32, 61], our study uniquely highlights the role of AMPs in this process. As a critical part of the innate immune response, AMPs, also known as host defense peptides, act to destroy invading pathogens using a variety of biological processes. AMPs, including CCL20, S100A8, SLPI and LCN2, use diverse mechanisms to defend against pathogens. For example, CCL20 promotes immune cell migration [62–64], S100A8 acts as a chemoattractant for neutrophils [65, 66], SLPI protects against neutrophil elastase, and LCN2 sequesters iron to inhibit bacterial growth. While discordant alterations in RNA and protein were noted for some AMPs, the effect of experimental timing on these results can’t be ruled out, as RNA and protein levels were assessed at the same time post bacterial exposure. However, notably, CCL20 was upregulated (RNA and protein) across all cell types in response to *G. vaginalis* and may play a critical role in recruiting immune cells to combat bacterial colonization. As a potent chemoattractant of lymphocytes and dendritic cells, CCL20 likely contributes to the recruitment of monocytes to defend against *G. vaginalis* colonization *in vivo*. Interestingly, CCL20 is the only chemokine that interacts with CC chemokine receptor 6 (CCR6), a property shared with the anti-microbial β-defensins. We have previously demonstrated that higher levels of b-defensin-2 were protective against preterm birth in the presence of specific anaerobes common to CST IV [4]. These two findings suggest that signaling through CCR6 by CCL20 and/or specific AMPs may be critical regulators of the host immune response to non-optimal bacteria. The role of CCL20 and/or other AMPs in limiting *G. vaginalis*-induced cell death and inflammation requires further investigation. Like CCL20, S100A8/A9 (also known as calprotectin) was increased after *G. vaginalis* exposure. In addition to neutrophil recruitment, S100A8/A9 acts to sequester metals/nutrients (calcium, iron, zinc, manganese) [67–69] as part of a process termed nutritional immunity in which metal-chelating host defense mechanisms are used to prevent infection [69, 70]. As most *Lactobacillus* species, except *L. iners*, require manganese for colonization [71, 72], it is possible that *G. vaginalis*-mediated increases in S100A8/A9 could act to limit *Lactobacillus* growth, thus potentially contributing to microbial dysbiosis in the cervicovaginal space. However, very little is known about the biological mechanisms regulating the effects of S100A8/A9 in the lower reproductive tract, and thus, elucidating the role of this AMP in modifying the cervicovaginal microbiome requires additional studies. It is biologically plausible that *G. vaginalis* induces diverse AMPs with opposing effects in the cervicovaginal environment, as evidenced by S100A8/A9’s ability to limit the bacterial colonization of optimal bacteria, while CCL20 stimulates the host immune response to reduce non-optimal bacterial colonization. Furthermore, specific AMPs may have multiple distinct functions in the cervicovaginal space. For instance, S100A8/A9 has been shown to promote leukocyte recruitment to initiate a host immune response [73], while simultaneously restricting nutrients essential for the growth of beneficial bacteria such as *Lactobacillus*. Interestingly, *L. crispatus* exposure downregulated the transcription of CCL20 and S100A8 suggesting that AMPs may help to promote *L. crispatus* growth. These findings provide further evidence for an intricate and complex relationship between the host and the vaginal microbiota.

In contrast to *G. vaginalis*, *L. crispatus* is considered an optimal bacterium that promotes reproductive health [2, 74–77]. Providing a plausible biological rationale for this protection, despite possessing a bacterial cell wall that should activate TLR-2, *L. crispatus* does not induce inflammation [22, 32] in part due to the presence of protective S-layer proteins [78]. However, the biological mechanisms underlying the beneficial properties of *L. crispatus* and other vaginal *Lactobacillus* spp. remain largely unknown, limiting the development of therapeutics that could leverage the beneficial properties of this bacteria. This study provides novel insight into these biological mechanisms and suggests that this protection may be mediated through epigenetic modifications. Specifically, *L. crispatus* culture supernatant modulates genes governing transcriptional and epigenetic regulation, leading to global reorganization of the epigenome in cervicovaginal epithelial cells. ATAC-seq analysis showed that exposure to *L. crispatus* culture supernatant reduced the number of open chromatin regions, suggesting a potential mechanism for increasing cervical epithelial cell resilience to infectious agents *(e.g., Chlamydia trachomatis*, HIV, HPV) [79, 80]. Consistent with our findings, a prior study demonstrated that *Lactobacillus* culture supernatants, specifically D-lactic acid, reduced *C. trachomatis* infection by modulating cell proliferation, a process essential for infection, via decreasing histone deacetylase 4 (HDAC4) and increasing histone acetylase EP300 gene expression [55]. While this previous study found that lactic acid induced these epigenetic modifications, further investigation is needed to identify additional metabolites or proteins present in *L. crispatus* culture supernatants that may alter the host epigenome. Since chromatin accessibility was altered in ectocervical cell lines, additional studies would be needed to determine whether similar chromatin alterations occur *in vivo* or in primary cervical cells. *L. crispatus*-mediated epigenome alterations in cervical epithelial cells supports a role for *L. crispatus* in protecting against microbial pathogens, vaginal infection, and even cervical cancer. [53]

Intriguingly, regions of differential chromatin accessibility did not overlap with known enhancer regions, likely due to the lack of ENCODE or ATAC profiles for primary cervical tissue for comparison. Further, the profiled cervical cancer specimens were derived from only four specimens and likely were a suboptimal reference for comparison to the ectocervical lines used in our study. This suggests that the identified differentially regulated regions are probably cell type-specific enhancers in ectocervical cells. The enrichment of intronic regions among differentially accessible sites provides evidence that *L. crispatus* may regulate isoform transcription through chromatin modulation. While, in this study, RNA sequencing could not address this hypothesis, future work employing long-read RNA sequencing may provide clarity [81]. Additionally, recent research has highlighted lactate, a precursor to acetyl-CoA, as both a precursor for histone acetylation and a direct modifier of histones via lactylation [82–85]. Histone lactylation could lead to increased gene expression putatively associated with more open chromatin. Further exploration of lactate’s role in chromatin accessibility and gene regulation could deepen our understanding of these processes. While we are unable to explain how differentially regulated sites contribute to gene regulation, disease gene enrichment analysis of transcription factors associated with the motifs at these sites indicate multiple pathologies related to women’s health, including fertility and endometriosis. These results point to an intriguing avenue of study to better understand the molecular underpinnings of these common but poorly understood reproductive disorders.

A limitation of this study is the focus on single vaginal bacterial species and strains. While whole microbiomes are needed to more accurately reflect the cervicovaginal microenvironment, focusing on individual bacteria allowed us to identify specific functional pathways underlying adverse outcomes. Future research should expand to include other high-risk anaerobic bacteria [4], such as *Sneathia*, *Mobiluncus* and *Prevotella* species, to elucidate their combined effects on cervicovaginal functions. Further, these studies will benefit from our foundational findings pointing to specific biological functions of both *G. vaginalis* and *L. crispatus* that could be leveraged to develop mechanistic hypotheses. Additionally, the inclusion of transcriptional profiles, including ATAC-seq data, from normal cervical and vaginal tissues in databases such as ENCODE, is critical for advancing [47] our understanding of host-microbial interactions in the reproductive tract and their role in reproductive outcomes.

In summary, this study identifies novel transcriptional and epigenomic pathways altered by common vaginal bacteria, highlighting the molecular complexity of host-microbial interactions in the cervicovaginal environment and their potential contributions to reproductive health and disease. Using unbiased sequencing approaches, we demonstrated that *G. vaginalis* activates the innate immune response, whereas *L. crispatus* modulates transcription and chromatin accessibility. Additionally, we found that these bacteria alter the transcriptional and epigenomic landscapes in distinct ways across the different epithelial surfaces in the lower reproductive tract. Collectively, our findings highlight potential therapeutic targets: 1) modulating the inflammatory response associated with *G. vaginalis* to mitigate STIs, bacterial vaginosis and preterm birth, and 2) leveraging *L. crispatus*-mediated epigenetic changes to strengthen cervicovaginal epithelial barriers against viral infections such as HPV. Continued investigations into host-microbial interactions in the female reproductive tract hold great promise for optimizing reproductive health.

## Materials and Methods

### Cell Culture

Ectocervical (Ect/E6E7, ATCC# CRL-2614) (Ecto), endocervical (End1/E6E7, ATCC# CRL-2615) (Endo) and vaginal (VK2/E6E7, ATCC# CRL-2616) (VK2) human epithelial cell lines (American Type Culture Collection, Manassas, VA) were cultured in Keratinocyte-Serum Free Media (KSFM) supplemented with 0.1 ng/mL epidermal growth factor and 50 μg/mL bovine pituitary extract (Gibco, Life Technologies), 100 U/mL penicillin, and 100 μg/mL of streptomycin at 37°C in a 5% CO_2_ humidi ed incubator.

### Bacterial Cultures and Preparation of Bacteria-Free Supernatants

Human clinical isolates of *L. crispatus* (ATCC 33197) or *G. vaginalis* (ATCC 14018), were obtained from the American Type Culture Collection (Manassas, VA). *G. vaginalis* was grown on Tryptic Soy Agar with 5% Sheep Blood plates (Hardy Diagnostics) and *L. crispatus* was grown on De Man, Rogosa and Sharpe agar (Fisher Scientific); both strains were grown in New York City III (NYCIII) broth. Bacteria were grown at 37°C in an anaerobic glove box (Coy Labs, Grass Lake, MI).

For each experiment the following bacterial growth protocol was followed: *L. crispatus* and *G. vaginalis* glycerol stocks were streaked on agar plates, as well as into broth tubes and grown overnight. The broth starter cultures were diluted to an optical density of 0.2 and then used to inoculate 20ml working cultures, which were grown for 20 hours (*G. vaginalis*) to 48 hours (*L. crispatus*) prior to use in experiments. Bacterial densities of the working cultures were estimated the day of the experiment based on optical density readings at 600 nm using an Epoch2 plate reader (Biotek, Winooski, VT), and the appropriate volume was centrifuged at 13,000 × *g* for 3 min. To obtain bacteria-free culture supernatants, the working cultures were centrifuged at 13,000 × *g* for 3 min and the supernatant was filtered through a 0.22 μm filter (Fisher Scientific) to remove any remaining live bacteria. Bacteria-free culture supernatants were diluted to 1% v/v in the appropriate cell culture media without antibiotics.

### In vitro Epithelial and Immune Cell - Bacteria Interactions

Ectocervical, endocervical and vaginal cells were plated at 1.5 × 10^5^ cells/well in twenty-four well plates containing KSFM without antibiotics. The next day, the cells were exposed to 1% (v/v) *L. crispatus* or *G. vaginalis* bacteria-free culture supernatants (generated from a 1×10^7^ CFU/mL culture) for 24 hr. Bacteria-free culture supernatant percentage was based on a dose response (1% vs 10%) (Supplemental Fig. 7). For cells exposed to 1% bacteria-free culture supernatants from *L. crispatus*, KSFM media was supplemented with 50mM HEPES and sodium bicarbonate (3000 mg/L total concentration) to bring the pH of the media up to a physiological level (7.2). For all supernatant experiments, cells were also exposed to 1% (v/v) NYCIII bacterial growth media alone (diluted in KSFM) to determine any baseline effects of the bacterial growth media on the outcomes of interest. 1% NYCIII (NYC) acted as the control for all bacteria-free culture supernatant exposures. At the end of each experiment, cell culture media was collected for cell death (supplemental methods), ELISA assays and/or the cells were collected in Trizol (Invitrogen, Thermo-Fisher Scientific) for RNA extraction.

### RNA Sequencing and Analysis

RNA was extracted from ectocervical, endocervical and vaginal cells after exposure to culture supernatants from *L. crispatus* and *G. vaginalis* (n = 3/treatment group) collected in Trizol using the Qiagen-RNeasy Plus Mini kit by the Penn Next-Generation Sequencing Core. The resulting cell death in these samples is shown in Supplemental Fig. 8. Despite some observed cell death following bacteria supernatant exposure, the resulting RNA had RIN values > 9. Illumina sequencing libraries were prepared using the Illumina TruSeq mRNA stranded library prep kit according to the manufacturer recommendations. The resulting libraries had an average molarity of 69 nM +/1 27 nM. Libraries were sequenced to a median depth of 41 million 100 bp single reads on an Illumina NovaSeq 6000. Transcript quantification from RNA-seq data was performed using Salmon and release 38 (GRCh38.p13) of the human genome [86, 87]. Several Bioconductor packages in R were used for subsequent steps [88, 89]. The output was annotated and summarized using tximeta and further annotation was completed with biomaRt [90, 91]. Principle Component Plots (PCA) were created using pcaExplorer [92, 93]. The normalizations and statistical analyses were done with DESeq2 [94]. Heatmaps for anti-microbial peptides were created using “pheatmap” in R (version 4.1.2). The full RNA-seq dataset was submitted to Gene Expression Omnibus (accession GSE234837).

### RNA-seq Pathway Analysis

*PathfindR* (v. 1.64) was used for pathway enrichment analysis using Gene Ontology terms (version from 2022–11-03) (https://github.com/egeulgen/pathfindR and https://release.geneontology.org/) [34, 35]. Upregulated and downregulated genes were grouped together for each comparison. The enrichment threshold was set at an FDR of 0.05, with a significant gene threshold of 0.02. A heatmap for enrichment scores for each comparison was created by first calculating and aggregating term scores for each sample included for each comparison and then averaging the scores across all compared samples as previously described [34]. *ComplexHeatmap* package in R (v. 2.14.0) was then used to visualize the comparison of GO term analysis (rows) for all the comparisons (columns) [37]. Rows were clustered by the “complete” method with a kmeans = 5. A word cloud was used to represent the most significant recurring pathways in a cluster. Generic terms or single letters were excluded from word cloud (“pathway”, “cellular”, “regulation”, “positive”, “negative”, “cell”, “complex”, “process”, “factor”, “activity”, “protein”, “dna”, “rna”, “levels”, “binding”, “response”, “signaling”, “receptor”, “production”, “t”, “ii”, “p”, “g”, “c”, “via”, “class”).

### ELISA

Ectocervical, endocervical or vaginal, cells were cultured in 24-well plates and exposed to bacterial culture supernatants as stated above. Anti-microbial peptides, CCL20, SLPI, LCN2, S100A8/A9, were measured in cell culture media after 24 hours of exposure (n = 3/group with n = 3 technical replicates per experiment). The expression of these analytes was measured by a ligand-specific commercially available ELISA kit that utilizes a quantitative sandwich enzyme immunoassay technique using reagents from R&D Systems (Minneapolis, MN).

### ATAC-seq Nuclei Extraction, Tagmentation, Purification and Library Amplification

ATAC-seq was performed on ectocervical, endocervical and vaginal cells after exposure to *L. crispatus* bacteria-free supernatants (n = 3/treatment group). ATAC-seq libraries were generated using the ATAC-seq Kit from Diagenode (Diagenode, A Hologic Company) according to manufacturer instructions. Briefly, nuclei were extracted from 50,000 cells. Tagmentation was completed by resuspending the isolated nuclei in transposase reaction mix and the samples were purified using the kit’s provided columns. Following purification, library fragments were amplified by PCR according to the manufacturer recommendations. Unique Dual Indexes Primer Pairs were incorporated for multiplexed sequencing. To reduce amplification bias, after the first 5 cycles of the PCR reaction, qPCR was used to determine how many additional cycles were needed to produce enough library to meet the required amount for sequencing. For this, an aliquot of the PCR reaction was added to Sybr Green and amplified for 20 cycles. Libraries were amplified for a total of 11–13 cycles (with one library requiring 17 cycles for amplification). Final libraries were purified using bead purification (Beckman Coulter), then assessed for size distribution and concentration using a BioAnalyzer High Sensitivity DNA Kit (Agilent Technologies). The resulting libraries were pooled. The pool was diluted to 2 nM, denatured, and the 13 libraries were loaded onto an S1–100 (2×50) flow cell on an Illumina NovaSeq 6000 (Illumina, Inc.) according to the manufacturer’s instructions. The average read number per sample was 50M+/− 20%. De-multiplexed and adapter-trimmed sequencing reads were generated using bcl2fastq. The full ATAC-seq dataset was submitted to Gene Expression Omnibus (accession # GSE233444).

### ATAC-seq Mapping and Peak Calling

ATAC-seq data analysis was adapted from a previously published approach using PEPATAC (v. 0.10.3) [41]. Peaks for each cervicovaginal line were called separately to allow for cell type specific differences in chromatin accessibility pattern. In brief, raw FASTQ files were processed and mapped to release 38 (GRCh38.p13) of the human genome using the PEPATAC pipeline [95]. Reads were trimmed with *skewer* and then aligned with *bowtie2* using default settings [96, 97]. Duplicate reads were removed using *samblaster* [98].

An iterative overlap peak calling strategy on fixed-sized peaks of 501 bp was used to de ne a set number of peaks for each cell type for downstream differential accessibility comparison [41]. First, for each biological replicate, MACS2 was used to call peaks with the parameters as follows: --peak-type fixed -- extend 250 [99]. Biological replicates of each treatment and then both treatments together from each cell type were merged using an iterative overlap approach previously described [41, 49]. Blacklisted regions were excluded from called peaks (accessed 4 November 2022 at https://github.com/Boyle-Lab/Blacklist) [100].

### ATAC-seq Peak and Differential Accessibility Analysis

Peak location was annotated with *CHIPseeker* (v 1.30.3) [101]. Counts for peaks were calculated using *Rsubread* (v. 2.8.2)[102]. We determined the differential accessibility of peaks between treatments with *DESeq2* (v. 1.34.0)[103]. We compared *L. crispatus* culture supernatant treated to NYCIII media controls for each cell type. A Wald test was used to determine significance. A peak was defined as statistically significant in differential accessibility if |log2foldchange| > 1 and FDR < 0.05. We utilized the R package *rGREAT* (v. 1.99.0) for the nearest gene analysis to access the Genome Regions Enrichment of Annotations Tool (GREAT) web service [44–46]. For GREAT, we used the parameters for “the two closest genes” to a differential accessible site as it is frequently not the closest genes that is differentially regulated.

Motif analysis was performed using Simple Enrichment Analysis version 5.5 as part of the MEME Suite (https://meme-suite.org/meme/tools/sea) [104, 105]. Differentially accessible sites were inputted, and the CIS-BP 2.0 motifs database were used for the query [106]. Gene-disease enrichment analysis was performed using *disgenet2R* (v. 0.99.2, https://www.disgenet.org/) [107]. Random gene lists were generated for comparison by sampling 497 transcription factors from the CIS-BP 2.0 database to ascertain the baseline disease enrichment bias of the database.

### ATAC-seq Chromatin Accessibility Visualization

*EasSeq* (v1) was utilized to visualize the data [108]. Biological replicates of BAM files were pooled for quantification of specific regions. Quantile normalization was used for counts per region for visualization to minimize bias from sequencing depth. Calculation of overlap performed both by any amount of overlap and the exact overlap of base pairs between all comparisons. Random regions for comparisons to differentially accessible regions or all ectocervical open chromatin regions were generated by Regulatory Sequence Analysis Tools matched for each cell type by number of fragments, fragment size, and GC content (random genome fragments tool; http://rsat.sb-roscoff.fr/) [109].

ENCODE datasets for all human enhancer-like sequences (ELS, defined as high DNAse-seq signal and high H3K37me3), or tissue-specific regulators were obtained from https://screen.encodeproject.org/ [47, 48]. For uterus and vaginal specimens, “Low-DNase” were filtered out to enrich for sites that had any evidence of potential enhancer or regulator activity. However, strict enhancer-like signature criteria could not be applied because all sequencing modalities were not available for all the samples. Primary cancer cell data sets were obtained from supplemental of published ATAC profiling [49]. Chi square analysis of number of overlapping sites was performed by Graph Pad.

### Statistical Analysis

Statistical analyses were performed for all experiments (except for RNA or ATAC sequencing, statistical analysis is described above for each) with the GraphPad Prism Software (Version 9.0, San Diego, CA). For data that were normally distributed (as assessed by Shapiro-Wilk test), one-way analysis of variance (ANOVA) was performed. If statistical significance was reached (p < 0.05), then pair-wise comparison with a Tukey post hoc test was performed for multiple comparisons. If data were not normally distributed, then the non-parametric Kruskal-Wallis test was used and pairwise comparison was done using Dunn’s multiple comparison test. Chi test for trend was utilized to compare overlaps of indicated ectocervical peaks with the number of a random set of sites matched for size and CG content.

## Data Availability

The full RNA-seq dataset was submitted to Gene Expression Omnibus (accession # GSE234837). The full ATAC-seq dataset was submitted to Gene Expression Omnibus (accession # GSE233444). All other data is provided within the manuscript or supplementary information files.
